# Estimation of methane greenhouse gas emissions from livestock in Egypt during 1989 to 2021

**DOI:** 10.1038/s41598-024-63011-0

**Published:** 2024-07-01

**Authors:** Mona Maze, Mohamed Omar Taqi, Rania Tolba, Ahmed A. A. Abdel-Wareth, Jayant Lohakare

**Affiliations:** 1 Central Laboratory for Agricultural Climate (CLAC), Agricultural Research Center (ARC), Giza, 12411 Egypt; 2 Agricultural Economics Research Institute, Agricultural Research Center (ARC), Giza, 12411 Egypt; 3 Department of Animal and Poultry Production, Faculty of Agriculture, South Valley University, Qena, 83523 Egypt; 4 Cooperative Agricultural Research Center, Prairie View A&M University, Prairie View, TX 77446 USA

**Keywords:** Climate change, Livestock, GHGs, Methane, Climate sciences, Environmental sciences, Environmental social sciences

## Abstract

This study investigates methane emissions from the livestock sector, representing by enteric fermentation and manure management, in Egypt from 1989 to 2021, focusing on spatial and temporal variations at the governorate level. Utilizing IPCC guidelines and emission factors, methane emissions were estimated for dairy and non-dairy cattle, buffalo, sheep and goat, poultry, and other livestock categories. Results reveal fluctuating emission patterns over the study period, with notable declines in certain governorates such as Kafr El-Sheikh and Red Sea, attributed to reductions in livestock populations. However, increasing trends were observed overall, driven by population growth in other regions. Hotspots of methane emissions were identified in delta governorates like Behera and Sharkia, as well as agriculturally rich regions including Menia and Suhag. While livestock populations varied between regions, factors such as water availability, climatic conditions, and farming practices influenced distribution. Notably, cluster analysis did not reveal regional clustering among governorates, suggesting emissions changes were not dependent on specific geographic or climatic boundaries. Manure management accounted for only 5–6% of total emissions, with emissions at their lowest in the last three years due to population declines. Despite the highest livestock populations being sheep and goats, emissions from enteric fermentation and manure management were highest from buffalo and cattle. This study underscores the importance of accurate data collection and adherence to IPCC recommendations for estimating GHG emissions, enabling the development of targeted mitigation strategies to address climate change challenges in the livestock sector.

## Introduction

Over the past three decades, the global population has experienced exponential growth, precipitating a corresponding surge in the livestock industry to meet the escalating demand for meat consumption^1^, and the consumption of natural resources as well, where for livestock production nearly 8% of freshwater and 30% of the world's land were utilized^2–4^. Furthermore, the global population has been predicted in the year 2050 to reach 9.5 billion people, which will lead to an increase in the produced animal protein to ~ 70%^4^*,* and therefore a higher consumption of natural resources that could lead to a series of environmental concerns^5,6^.

Global warming is caused by the emissions of greenhouse gases (GHG). These pollutants contribute to the increase of temperature and extreme weather events, which have adverse effects on animal metabolism, health, reproduction, and productivity. On the other hand, the expected increased demand for animal-origin products in the coming years will increase the livestock census and consequently GHG emissions^4^. The twin challenges of increasing productivity to meet demand and reducing emissions to meet climate commitments represent a serious concern in livestock production^7,8^. The livestock sector is a main source of non-CO_2_ GHG emissions (CH_4_ and N_2_O), as well as the whole agriculture sector^9–13^, with a large potential for emissions reductions^14–18^, where livestock farming is estimated to contribute to about 18% of total global greenhouse gas (GHG) emissions, considering direct and indirect land use^17,18^. Not only the livestock sector has an influence on climate change, but also on the biodiversity loss and degradation of land and freshwater^10,19^.

According to the rapid increase in anthropogenic emissions^20,21^, the atmospheric CH_4_ concentration has increased from 1644.85 ppb in 1984 to 1911.80 ppb in 2022^22^. Livestock production contributes to about 37% of methane (CH_4_) emissions and about 65% of nitrous oxide (N_2_O) emissions worldwide^17,23^, where only beef cattle have accounted for approximately one-third of the world anthropogenic CH_4_ emissions^4^ and almost three quarters to total livestock emissions^9^. Therefore, much attention has been paid to the development of a low-carbon emissions strategy for beef production^24,25^.

The GHG emissions from the livestock sector came from the subcategories enteric fermentation and manure management. Ruminants are responsible for the largest share of enteric fermentation and manure production^4,26^, although ruminant farming systems vary depending on physical conditions such as climate, soil type, altitude, landscape^27,28^, species (cow, goat, sheep), and production purpose (dairy or meat)^29^. The process of anaerobic fermentation in the rumen and large intestines is responsible for the release of enteric methane into the atmosphere as the ruminant animals respire methane gas from the mouth and nostrils^30^. In addition, livestock excreta (manure and urine) and its subsequent handling and management practices contribute to on-farm GHG emissions. Manure management practices differ across livestock farming systems, manure form (solid or liquid), and quantities produced among other factors^31^. A complex of microbial activities and chemical processes dictated by the prevailing anaerobic conditions result in a fluctuating production of nitrous oxide and methane from livestock excreta^32,33^.

Situated at the juncture of Africa and Asia, Egypt's unique geographical setting is flanked by the Mediterranean Sea and the Red Sea. The Nile River, coursing from south to north, nourishes the fertile Nile Valley and Delta regions, supporting the bulk of Egypt's population and economic endeavours ^34,35^. Moderate temperatures and sporadic rainfall in these regions create favourable conditions for cultivating essential crops such as wheat, rice, corn, and cotton. In contrast, the vast Western and Eastern Deserts, spanning 96% of Egypt's landmass, offer minimal habitable terrain, sustaining sparse vegetation and agriculture reliant on traditional irrigation methods Despite arid weather patterns characterized by scorching summers and mild winters, agriculture remains a cornerstone of the economy. Livestock rearing, including water buffalo, cattle, goats, sheep, and poultry, supplements crop cultivation efforts. Even in desert regions, nomadic herders tend to camels, goats, and sheep, sustaining livelihoods amid harsh conditions. Modern practices like drip irrigation and resilient crop varieties are being embraced to bolster agricultural productivity and mitigate the impacts of water scarcity and climate variability^34–36^.

The agriculture, forestry, and land use (AFOLU) sector in Egypt contributed to 14.9% (48,390 Gg CO_2_ equivalent) of the total GHG emissions in 2015, where the livestock represented 34% of them. The enteric fermentation and manure management shared the AFOLU non-CO_2_ emissions with 65% and 35% respectively^37^. The total emissions of CH_4_ from enteric fermentation revealed 394 Gg for the year 2005. Buffalo was the key source of CH_4_ emissions from enteric fermentation, of total emissions of 213.68 Gg, accounting for 48% whereas cattle was the second key source with total emissions of 154.44 Gg. While total CH_4_ emissions from manure management revealed 30.46 Gg for the year 2005. Likewise, buffalo was the key source of CH_4_ emissions from manure management, with total emissions of 19.03 Gg (64%), and for cattle, it is 7.22 Gg in 2005^38^.

This investigation aimed to: (1) generate methane emission inventories from the large livestock sector at the governorate level in Egypt; (2) understand the spatial and temporal dynamics of methane emissions from the livestock sector during 1989–2021 and identify emission hotspots across different governorates. Methane emissions were estimated for the different livestock categories in Egypt, for examining spatiotemporal patterns by governorates, following IPCC guidelines^39^.

## Data and methods

### Study area description

**Figure 1:**
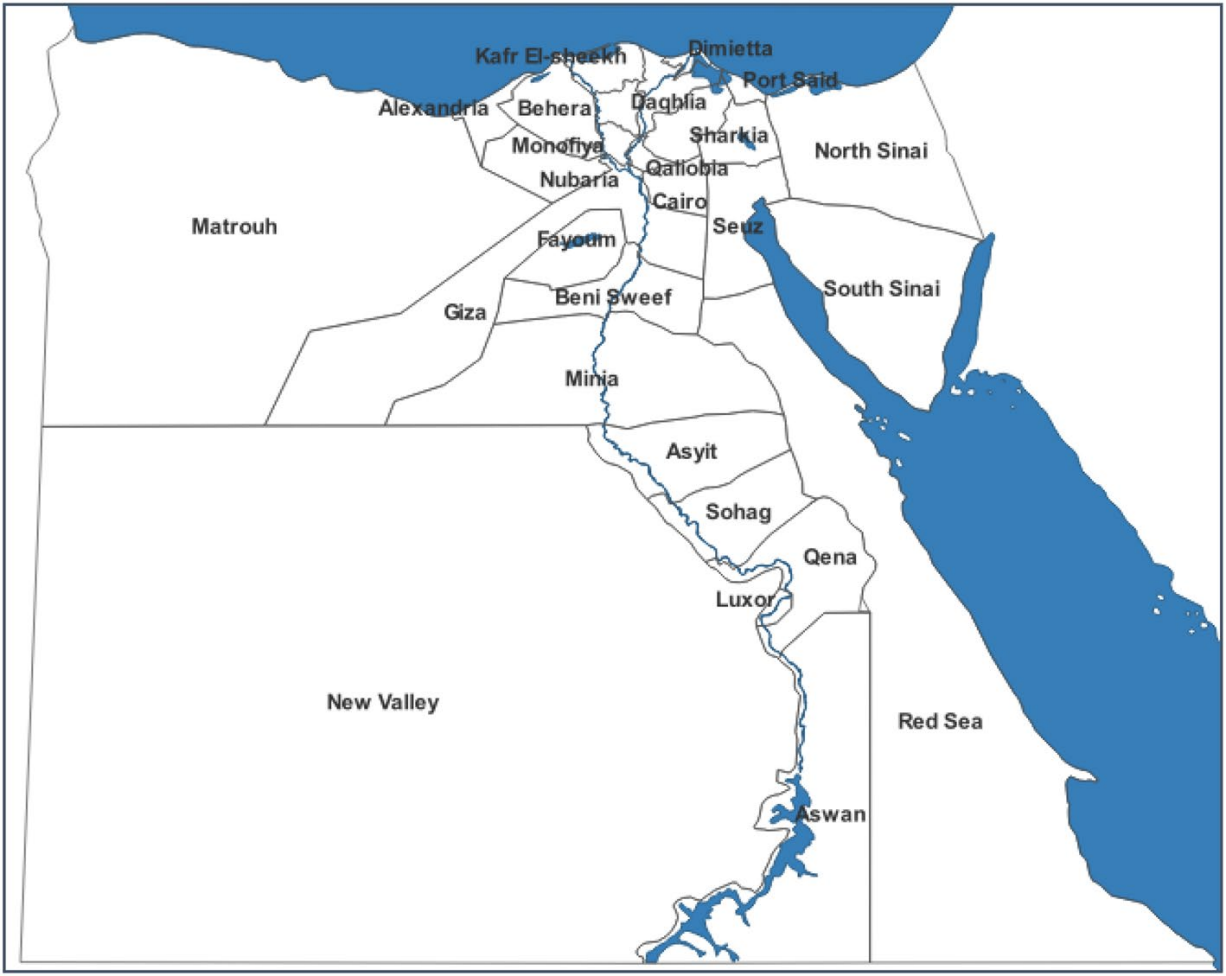
Map of Egypt with its governorates and regions. Digital Map was generated using GIS software, specifically QGIS 3.20 (https://www.qgis.org/en/site/forusers/download.html#).

Egypt's unique geographical location at the intersection of Africa and Asia, bordered by the Mediterranean Sea and the Red Sea, contributes to its diverse climate and agricultural landscape. The country's agricultural heartland is the Nile Valley and Delta regions, sustained by the life-giving waters of the Nile River. This fertile area, referred to as "inside the Valley," comprises less than 5% of Egypt's total land area. The remaining vast expanses of desert are termed "outside the Valley". Egypt is administratively divided into 27 governorates, categorized into four climatic and geographical regions. The first three regions, representing Lower, Middle, and Upper Egypt, are situated within the Nile Valley. The remaining governorates are located outside the Valley. Notably, Nubaria is considered a governorate outside the Valley, although geographically part of Bahera governorate, as it lies entirely in the desert (Fig. 1, Table 2).

### Livestock population

Livestock categories are broadly classified into six sections: dairy cattle, non-dairy cattle, buffalo, sheep and goats, poultry, and others, encompassing miscellaneous animals such as swine, horses, camels, mules, and asses. Annual population data for each livestock category in Egypt from 1989 to 2021 were sourced from the statistical annual reports of the Central Agency for Public Mobilization and Statistics (CAPMAS)^40^ and the Economic Affairs Sector (EAS)^41^. For missing years (1992, 1994, 1996, 2006, and 2008), estimates were derived by averaging data from the preceding and succeeding years.

### Livestock production system

Egypt's livestock production system exhibits a predominance of local and mixed breeds, constituting approximately 92% of the cattle population, with the remaining 8% comprising foreign breeds. Livestock farming is predominantly concentrated in the Nile Delta, with local and mixed cattle accounting for 55% of the population, while foreign breeds are more prevalent in desert areas, representing 30% of the total. Cattle breeds vary across regions, with the Nile Delta hosting Bahari, Menoufi, Damietta, Holstein, Brown Swiss, and Friesian breeds, while Central and Upper Egypt harbor Saadi, Abondance, Trantiz, and other breeds. Female cattle represent around 66% of the local and mixed breed population and 68% of foreign breeds. Buffalo farming exclusively involves local breeds, with females comprising approximately 71% of the herd. Sheep and goat breeds, including Barakat, Rahmani, Falahi, Osimi, Sinai Black, and Egyptian Nubian, are distributed across different governorates. Livestock nutrition predominantly relies on concentrated feed, silage, maize, berseem in winter, and corn and fodder in summer. Grazing primarily occurs in the Eastern and Western deserts, particularly for sheep and goats^41,42^.

### Livestock management system

Livestock management in Egypt encompasses semi-intensive production programs prevalent in the Delta and Nile Valley, characterized by small-scale holdings housing 1 to 2 buffalo or cattle. These holdings constitute approximately 85% of Egypt's livestock and primarily feed on cultivated green fodder year-round, supplemented with crop residues, industrial by-products, and concentrated feed. Intensive programs focus on milk production or fattening, while non-intensive ones in desert areas rely on natural pastures supplemented by barley and feed. Data indicates the presence of about 15,000 fattening farms, with small farms (less than 25 head) accounting for roughly 48% and medium-sized farms (less than 50 head) around 32%. Dairy farms number around 10,000, with small farms making up about 54% and medium-sized farms approximately 24%. Manure management practices in Egypt include organic compost production and biogas units^41,43^.

### Methane emissions calculation

Estimating methane (CH_4_) emissions from enteric fermentation and manure management entails specific procedures and methods in accordance with the 2019 refinement of the IPCC 2006 guidelines (2019). The estimation process involves gathering livestock population data categorized by animal subgroups, estimating emission factors for each subgroup or utilizing default emission factors, and multiplying these factors by their respective populations^39,44^.

### Emission factors (EFs)

Livestock CH_4_ emissions encompass enteric fermentation and manure management. Due to the unavailability of country-specific emission factors, CH_4_ emissions are estimated using the Tier 1 methodology based on EF values reported in the 2019 refinement of IPCC 2006 guidelines^39^. These EF values are contingent on factors such as animal type, climate zone (temperate), geographic region (Africa and Middle East), and productivity system (Low productivity systems). Separate EFs are utilized for each category of cattle (non-dairy – dairy), buffalo, sheep, goats, camels, and horses to calculate emissions from enteric fermentation or manure management. The resulting methane emissions are presented in CO_2_ equivalent (CO_2e_) form. All the used factors in the following equations are listed in Table 1.

**Table 1:** Emission factors of emitted methane from livestock sector (kg CH_4_ head^−1^ year^−1^)*.

Category	Enteric fermentation EF (kg CH_4_ head^−1^ yr^−1^)^1^	VS excretion rate kg VS (1000 kg animal mass)^−1^ day^−1^)^1^	Typical animal mass (TAM) (kg animal^−1^)^1^	Manure Management (G CH_4_ kg VS^−1^)^2^	Animal waste management system (AWMS)^2^
Dairy Cattle	62	11.8	270	3.5, 1.3, 0.4, 9.5, 8.7	SS, DL, DS, B, BF
Non-dairy cattle	55	14.5	232	3.5, 1.3, 0.4, 9.5, 8.7	SS, DL, DS, B, BF
Buffalo	67	13.5	381	3.5, 1.3, 0.4, 9.5, 8.7	SS, DL, DS, B, BF
Sheep	5	8.3	31	1.3, 0.6	DL, PRP
Goat	5	10.4	24	1.3, 0.6	DL, PRP
Camels	46	11.5	217	2.1, 0.6	DL, PRP
Horses	18	7.2	238	2.6, 0.6	DL, PRP
others	10	7.2	130	2.6, 0.6	DL, PRP
Poultry	–	16.5	0.7	2.4	All systems

^1^Middle east, low productivity systems.

^2^Temperate zone, and low productivity.

*According to 2019 refinement of IPCC 2006 guidelines^39^.

### IPCC equations

Methane emissions from enteric fermentation are calculated using the equation:

$${N}_{T}=Day{s\_alive}* \left(\frac{NAPA}{365}\right)$$

where, N_T_ is the no. of head of animal in T country, Days_alive is the average age of the animal, and NAPA is number of animals produced annually.

CAPMAS reported that the production of broiler chickens is till 60 days old, while the breeding and production of broiler chickens is till 30 days.

The methane emissions from enteric fermentation were calculated as:

$${E}_{T}= {\sum }_{(P)}{EF}_{\left(T,P\right)}* \left(\frac{{N}_{(T,P)}}{{10}^{6}}\right)$$

where, ET represents methane emissions from Enteric Fermentation in animal category T (Gg CH_4_ yr^−1^), EF_(T,P)_ denotes the emission factor for the defined livestock population T and productivity system P (kg CH_4_ head^−1^ yr^−1^), and N_(T,P)_ signifies the number of head of livestock species/category T in the country classified as productivity system P.

Methane emissions from manure management are calculated using the equation:

$${CH}_{4(mm)}= \left[{\sum }_{T,S,P}\left({N}_{(T,P)}* {VS}_{\left(T,P\right)}* {AWMS}_{\left(T,S,P\right)}* {EF}_{(T,S,P)}\right)/1000\right]/{10}^{6}$$

where, CH_4(mm)_ denotes CH_4_ emissions from Manure Management in the country (kg CH_4_ yr^−1^), VS_(T,P)_ represents the annual average Volatile Solids excretion per head of species/category T, for productivity system P (kg VS animal^−1^ yr^−1^), AWMS_(T,S,P)_ signifies the fraction of total annual VS for each livestock species/category T managed in manure management system S, and EF_(T,S,P)_ indicates the emission factor for direct CH_4_ emissions from manure management system S, by animal species/category T, in productivity system P (g CH_4_ kg VS^−1^).

Annual Volatile Solids (VS) excretion is calculated using the equation:

$${VS}_{(T,P)}= \left({VS}_{rate(T,P)}* \frac{{TAM}_{(T,P)}}{1000}\right)*365$$

where, VS_rate(T,P)_ represents the default VS excretion rate for productivity system P (kg VS (1000 kg animal mass)^−1^ day^−1^), and TAM_(T,P)_ denotes the typical animal mass for livestock category T, for productivity system P (kg animal^−1^).

Methane emissions from enteric fermentation and manure management are then converted from CH_4_ to CO_2e_ using the conversion factor:

$$emissions\left({CO}_{2e}\right)=emissions\left({CH}_{4}\right)*25$$

### Digital map generation

To generate spatial maps of Egypt at the governorate level, GIS software, specifically QGIS 3.20 (https://www.qgis.org/en/site/forusers/download.html#) was utilized to visualize CH_4_ emissions data from livestock. These digital maps facilitate a comprehensive understanding of emission patterns across different regions. Upon generating methane emissions data, the information was used to construct a shapefile database of Egyptian governorates (Fig. 1), enabling classification and generation of digital maps.

### Data analysis

Linear regression analysis was conducted on enteric fermentation and manure management methane emissions across the studied period for each governorate to estimate the overall emission trend. The trend (slope) and p-value were calculated, where the trend indicates the total increase (+) or decrease (−) of emissions during the studied period. The p-value serves as a statistical measure used to validate a hypothesis against observed data. A statistically significant result of linear regression is indicated by a p-value of ≤ 0.05. Cluster analysis was performed on methane emissions to interpret changes, represented as the ratio of emissions in 2005 to 1989 and 2021 to 2005, expressed as percentages. The Ward method was employed, and the output was visualized using a hierarchical dendrogram and boxplot diagram.

## Results

### Livestock census

**Figure 2:**
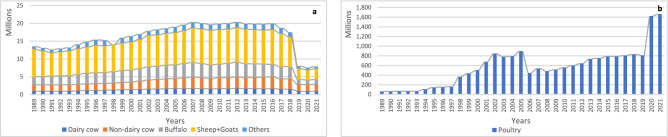
The livestock population during the period 1989 to 2021 categorized (**a**) dairy and non-dairy cattle, buffalo, sheep + goat and others; (**b**) poultry.

The evolution of the livestock population with all its categories is shown in Fig. 2. The livestock population (dairy and non-dairy cattle, buffalo, sheep and goats, and others) displayed a fluctuating trend over the years (Fig. 2a). From 1989 to 1991, there was a slight decrease in the total livestock population, with declines of 3.4% and 3.5%, respectively. However, from 1992 to 2016, there was a gradual increase in the livestock population, except for a slight decline in 1998, where the total population decreased by 7.5%. Notably, there was a significant decline in the 'others' category in 1998, reaching 91% compared to 1997. The peak years for the highest populations were 2007, 2012, and 2016, with around 20 million animals. After 2016, there was a slight descending trend observed in the total livestock population, with decreases of 6.2% and 6.5% in 2017 and 2018, respectively. A significant fall in the livestock population occurred in 2019, dropping by 54.4% compared to 2018. Subsequently, the livestock population remained relatively constant in 2020 and 2021. Around half of the total livestock population was represented by the sheep and goat categories.

On the other hand, the poultry population (Fig. 2b) exhibited a totally different pattern than the rest of the livestock population during the period from 1989 till 2021. The poultry population showed a wave-like pattern with bottom years, where the poultry population was low and started to gradually increase after reaching a peak year, followed by another wave. Each wave differed in its width and height. The bottom years were 1989, 2003, 2006, and 2008, with populations of 62.6563, 783.519, 444.922, and 483.847 million, respectively. While the peak years were 2002, 2005, 2007, and 2021, with populations of 847.994, 898.671, 537.966, and 1666.76 million, respectively. The last two years, 2020 and 2021, showed a significant increase compared to the other studied years, with approximately double the value of 2019.

### Methane emissions from enteric fermentation

**Figure 3:**
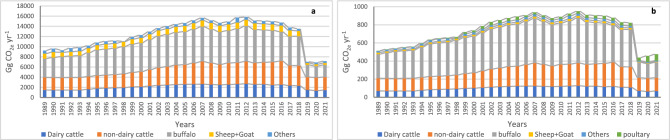
The enteric fermentation (**a**) and manure management (**b**) CH_4_ emissions (Gg CO_2e_ yr^−1^) categorized by animal type during the period 1989 to 2021.

A similar pattern to the livestock population appeared for its methane enteric fermentation emissions in Fig. 3a, where the enteric fermentation emissions calculations are mainly associated with the livestock population. The lowest emissions appeared in the years 2019, 2020, and 2021, with 7037.5, 6895.1, and 7162.8 Gg CO_2e_ yr^−1^, respectively, which is around half the emissions of the last year 2018. While the highest methane emitted from enteric fermentation was 15,661.4, 15,375.5, and 15,824.2 Gg CO_2e_ yr^−1^ in the years 2007, 2011, and 2012, respectively.

The calculated emitted methane from buffalo and cattle (dairy and non-dairy) were close to each other, ranging from 40 to 50% each, and a total of 82% to 90% of the total emissions. In the last three years, cattle emissions were higher than buffalo by about 20%. The rest of the categories had from 9 to 16% of the total enteric fermentation methane emissions.

### Methane emissions from manure management

The methane manure management emissions in Fig. 3b had a combined appearance of the livestock and poultry population figures, where all the animal categories, including poultry population, were counted for measuring the methane emitted from manure management. The highest emissions were recorded in 2007, 2011, and 2012, ranging from 938.5 to 950.7 Gg CO_2e_ yr^−1^. However, from 2019 to 2021, emissions ranged from 438 to 475 Gg CO_2e_ yr^−1^, which was around half the emissions of 2018.

The emissions from buffalo had the highest share, ranging from 47 to 58%, followed by cattle with around 40%. Together, buffalo and cattle contributed from 90 to 95% of the total emissions, while in the last three years, these shares changed to 88%, 81%, and 81%, respectively. Poultry emissions, although low initially (1%), increased significantly in the last three years, reaching a share of 8%, 8%, and 15%, respectively. The sheep, goat, and others categories represented 3% to 7% of the total methane emissions from manure management.

### Total methane emissions from the livestock sector in Egypt

**Figure 4:**
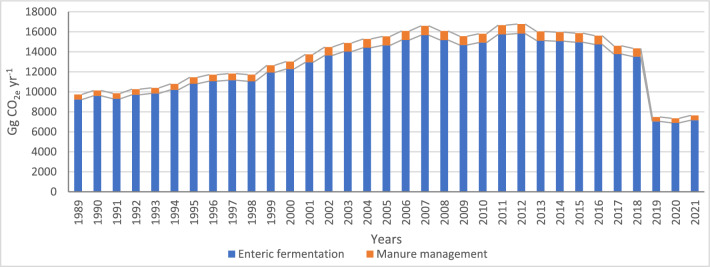
The methane total emissions from livestock (Gg CO_2e_ yr^−1^) including enteric fermentation and manure management operations during the period 1989–2021.

The total methane emissions of all livestock categories (Fig. 4) followed a similar pattern to methane enteric fermentation emissions, where methane manure management emissions represented from 5.2% in the early years to 6.2% in the last two years of the enteric emissions values. The lowest emissions appeared in the years 2019, 2020, and 2021, with values of 7475.6, 7352.2, and 7683.2 Gg CO_2e_ yr^−1^, respectively, while the highest emissions were noted in the years 2007, 2011, and 2012, with values of 16,599.9, 16,658.9, and 16,774.8 Gg CO_2e_ yr^−1^, respectively.

### Distribution of methane emissions from enteric fermentation in Egyptian governorates

**Figure 5:**
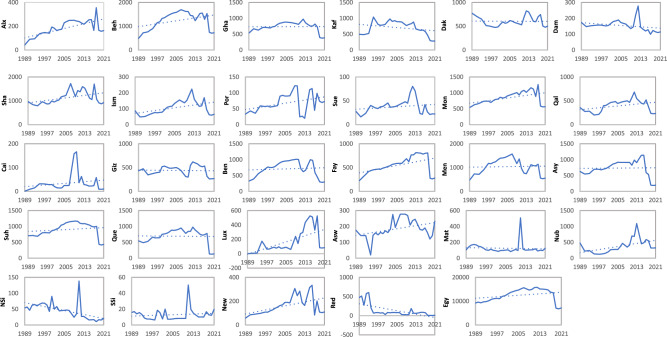
The evolution of the methane enteric fermentation emissions (Gg CO_2e_ yr^−1^) of livestock from the period 1989 till 2021 at the governorates level.

The distribution of methane emissions from enteric fermentation in the period 1989–2021 across different governorates and regions in Egypt is shown in Fig. 5. The emissions varied across governorates and regions, showcasing both positive and negative trends over the years. While some governorates experienced an increase in methane emissions from enteric fermentation, others saw a decrease. For example, Behera, Sharkia, and Monofia in Lower Egypt exhibited increasing trends, with peak emissions recorded in 2007, 2017, and 2018, respectively. On the other hand, Kafr El-Sheikh, North Sinai, and the Red Sea governorates showed decreasing trends, with notable declines in emissions over the studied period. Overall, the distribution of enteric fermentation emissions from 1989 to 2021 in Egypt's governorates demonstrated mostly lower emissions at the start of the period, followed by a gradual increase through the years. However, there were variations in the peak year emissions, and emissions decreased rapidly in some governorates, especially in the last three years of the study period.

The linear regression estimation of the enteric fermentation distribution (Table 2) indicated sometimes insignificant changing trends (slope) during the studied period for certain governorates. For example, Gharbia, Dakahlia, Damietta, Suez, Cairo, Giza, Asyut, Quena, Matrouh, and South Sinai showed insignificant changes in trend, with p-values greater than 0.05. Conversely, significant increases in trend were observed for Alexandria, Behera, Sharkia, Monofia, Qalubia, Fayoum, Luxor, Nubaria, and New Valley, with p-values less than 0.05. Meanwhile, Kafr El-Sheikh, North Sinai, and the Red Sea experienced significant decreases in trend, also with p-values less than 0.05.

**Table 2:** Slope and p-value of linear regression for methane emissions during 1989 to 2021 at the different governorates and regions in Egypt.

Governorates		Enteric fermentation (CO_2e_)		Manure management (CO_2e_)	
Name	Symb	*p-value*	*Slope*	*p-value*	*Slope*
Alexandria	Alx	< 0.05	4.986	< 0.05	0.304
Behera	Beh	< 0.05	15.308	< 0.05	1.09
Gharbia	Gha	> 0.05	0.142	> 0.05	0.118
Kafr El-Sheikh	Kaf	> 0.05	-6.245	> 0.05	-0.332
Dakahlia	Dak	> 0.05	-0.183	> 0.05	0.078
Damietta	Dam	> 0.05	-1.08	> 0.05	-0.03
Sharkia	Sha	< 0.05	13.234	< 0.05	0.965
Ismailia	Ism	< 0.05	2.219	< 0.05	0.152
Port Said	Por	< 0.05	1.248	< 0.05	0.083
Suez	Sue	> 0.05	0.322	> 0.05	0.023
Monofia	Mon	< 0.05	10.355	< 0.05	0.662
Qalubia	Qal	< 0.05	4.868	< 0.05	0.424
Cairo	Cai	< 0.05	0.848	> 0.05	0.058
Lower Egypt	LEg	> 0.05	46.013	< 0.05	3.594
Giza	Giz	> 0.05	-0.492	> 0.05	-0.043
Beni Swaif	Ben	> 0.05	2.068	> 0.05	0.149
Fayoum	Fay	< 0.05	9.666	< 0.05	0.625
Menia	Men	> 0.05	1.377	> 0.05	0.12
Middle Egypt	Meg	> 0.05	12.617	> 0.05	0.851
Asyut	Asy	> 0.05	0.564	> 0.05	0.011
Suhag	Suh	> 0.05	3.778	> 0.05	0.215
Quena	Que	> 0.05	-0.607	> 0.05	-0.001
Luxor	Lux	< 0.05	10.788	< 0.05	0.648
Aswan	Asw	< 0.05	2.508	< 0.05	0.14
Upper Egypt	UEg	> 0.05	17.028	> 0.05	1.013
Inside Valley	InV	> 0.05	75.654	> 0.05	5.457
Matrouh	Mat	> 0.05	-0.492	> 0.05	0.059
Nubaria	Nub	< 0.05	12.252	< 0.05	0.739
North Sainai	NSi	< 0.05	-1.56	> 0.05	-0.009
South Sainai	SSi	> 0.05	0.106	> 0.05	0.006
New Valley	New	< 0.05	4.238	< 0.05	0.229
Red Sea	Red	< 0.05	-11.253	< 0.05	-0.332
Outside Valley	OuV	> 0.05	3.278	< 0.05	0.691
Total Egypt	Egy	> 0.05	77.933	< 0.05	6.149

### Methane emissions from Egyptian governorates associated with manure management

**Figure 6:**
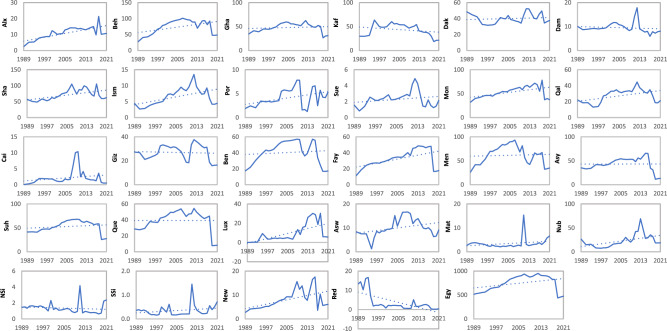
The evolution of the methane manure management emissions (Gg CO_2e_ yr^−1^) of livestock from the period 1989 till 2021 at the governorates level.

The pattern of methane emissions emitted from manure management during 1989–2021 at Egypt governorates and regions is shown in Fig. 6. The distribution of manure management methane emissions revealed a similar pattern to enteric fermentation emissions but with more than 10 times lower values. Many governorates presented insignificant increasing and decreasing trends during the studied period. The highest increase in the trend was observed in Behera, Sharkia, Monofia, Qalubia, Fayoum, Luxor, and Nubaria (Table 2).

### Methane emissions by Egyptian governorates from livestock

#### Population and emissions by governorates and regions

**Figure 7:**
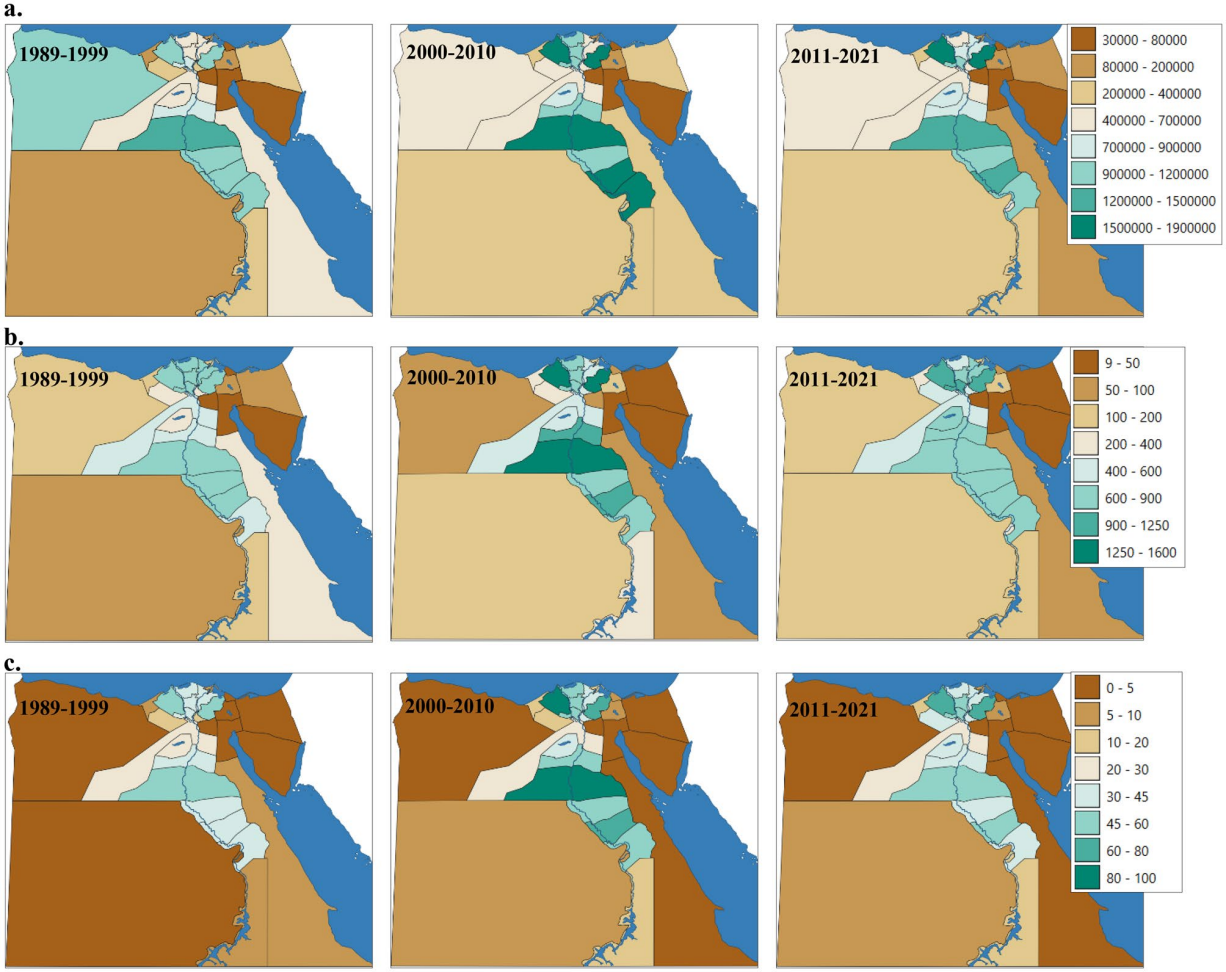
Maps of regional (governorates) distributions in Egypt for the three periods 1989 to 1999, 2000 to 2010 and 2011 to 2021 (the average of the periods): (**a**) livestock population (poultry is represented in thousands); (**b**) enteric fermentation methane emissions for all livestock categories (Gg CO_2e_ yr^−1^); and (**c**) manure management methane emissions for all livestock categories (Gg CO_2e_ yr^−1^). Digital Map was generated using GIS software, specifically QGIS 3.20 (https://www.qgis.org/en/site/forusers/download.html#).

The studied period (1989–2021) was divided into three average periods of 1989–1999, 2000–2010, and 2011–2021. Figure 7 presents the distribution of the total livestock population, methane enteric fermentation, and manure management emissions for each governorate and region. During the second average period (2000–2010), certain governorates recorded the highest total livestock population and methane emissions from both enteric fermentation and manure management. Notable examples include Menia, Behera, Sharkia, Suhag, and Quena. Conversely, the first average period (1989–1999) showed the highest values in outside Valley governorates, except for New Valley. Specifically, Menia, Behera, Sharkia, Suhag, and Quena demonstrated the highest livestock populations during the second average period, with respective values of 1,874,350, 1,819,082, 1,577,707, 1,523,635, and 1,500,791. In contrast, during the third average period (2011–2021), the highest populations were recorded in Sharkia, Behera, Menia, Suhag, and Quena, with respective values of 1,666,692, 1,501,126, 1,310,814, 1,296,682, and 1,004,007.

Concerning methane emissions, Behera, Menia, Sharkia, Suhag, and Monofia exhibited the highest enteric fermentation emissions during the second average period, with values of 1,159.8, 1,386.9, 1,254.0, 1,068.0, and 895.9 Gg CO_2e_ yr^−1^, respectively. During the third average period, the highest enteric fermentation emissions were observed in Behera, Menia, Sharkia, Suhag, and Monofia, with values of 1,235.8, 860.2, 1,222.7, 877.3, and 941.1 Gg CO_2e_ yr^−1^, respectively. Similarly, the highest manure management emissions during the second average period were recorded in Behera, Menia, Sharkia, Suhag, and Monofia, with values of 93.1, 82.7, 79.0, 62.4, and 56.8 Gg CO_2e_ yr^−1^, respectively. In the third average period, Behera, Menia, Sharkia, Suhag, and Monofia also had the highest manure management emissions, with values of 75.7, 50.8, 78.3, 51.2, and 59.3 Gg CO_2e_ yr^−1^, respectively.

#### Population and emissions share by animal category

**Figure 8:**
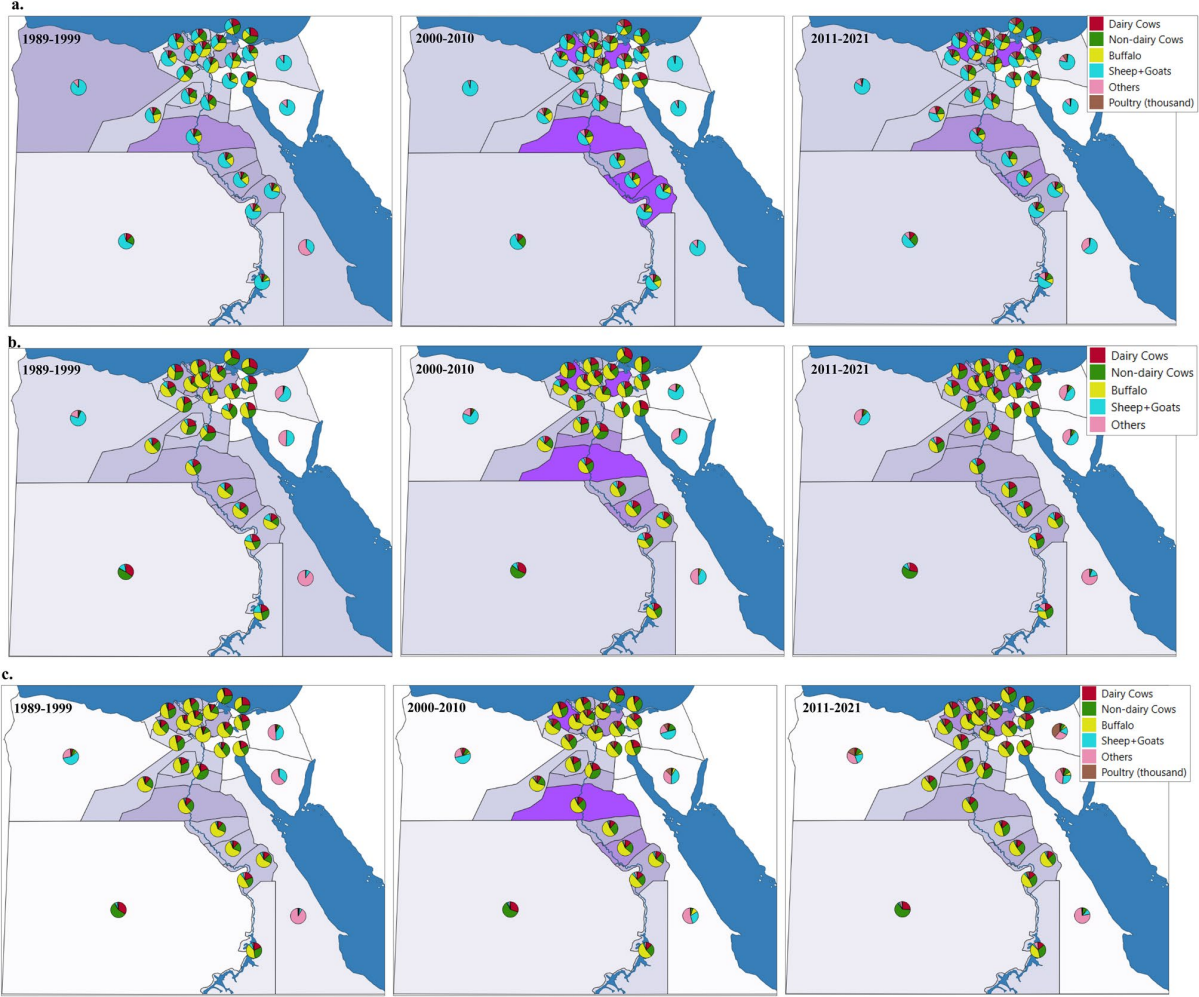
Maps of regional (governorates) distributions in Egypt for the three periods 1989 to 1999, 2000 to 2010 and 2011 to 2021 (the average of the periods): (**a**) categorized livestock population (poultry is represented in thousand); (**b**) enteric fermentation methane emissions for categorized livestock (Gg CO_2e_ yr^−1^); and (**c**) manure management methane emissions for categorized livestock (Gg CO_2e_ yr^−1^). The darker background of the governorate represents higher total value than lighter colour. Digital Map was generated using GIS software, specifically QGIS 3.20 (https://www.qgis.org/en/site/forusers/download.html#).

Figure 8 illustrates the share of each animal category of population and emissions for each governorate and region across the three average periods. Across the three average periods, the sheep and goat category consistently showed the highest share among the rest of the categories, both inside and outside the Valley regions. Inside the Valley, the share of the sheep and goat category ranged from 22 to 74% during the first, second, and third average periods. Conversely, outside the Valley, the contribution of the sheep and goat category dominated even more, with shares ranging from 38 to 95% during the same periods.

The buffalo and cattle (dairy and non-dairy) categories demonstrated similar contributions, ranging from 22 to 88% inside the Valley and from 0 to 61% outside the Valley across the three average periods. Meanwhile, the poultry category, absent from the enteric fermentation process, exhibited very low shares ranging from 3 to 13% inside the Valley and from 13 to 39% outside the Valley during the third average period, indicating its significant contribution to emissions from manure management.

### Emissions pattern throughout the investigation period

**Figure 9:**
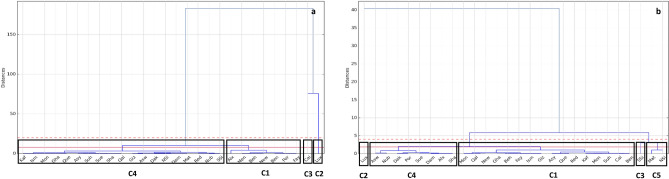
Clusters of CH_4_ emissions change (enteric fermentation and manure management) between the years 1989–2005 (**a**) and 2005–2021 (**b**) by the different governorates. The change was calculated by dividing the values of the recent year by the old year (2005/1989 and 2021/2005), expressed in %, C1–C5: clusters.

Governorates’ emission patterns differed from one to the other. For more understanding of the similarity of the governorates’ pattern, cluster analysis was applied on the calculated methane emissions from enteric fermentation and manure management processes in each governorate. Three years were selected: 1989, 2005, and 2021 to cover the whole studied period. The dendrogram explained the changes between the total methane emissions in 1989 and 2005 by dividing 2005 emissions by 1989 emissions, and secondly in 2005 and 2021 by dividing 2021 emissions by 2005 emissions (Fig. 9). The results of the clustering analysis of the first division in dendrogram resulted in four clusters (C1–C4), where C1 represented Alexandria, New Valley, Port Said, Beni Swaif, Behera, Fayoum, and Menia governorates, C2 represented Luxor governorate, C3 represented Cairo governorate, and C4 represented the rest of the governorates. The results of the clustering analysis of the second division in dendrogram gave five clusters, where C2 represented Luxor, C3 represented South Sinai, C4 represented Aswan, Nubaria, Dakahlia, Port Said, Sue, Damietta, Alexandria, and Sharkia, C5 represented North Sinai and Matrouh and C4 represented the rest of the governorates.

**Figure 10:**
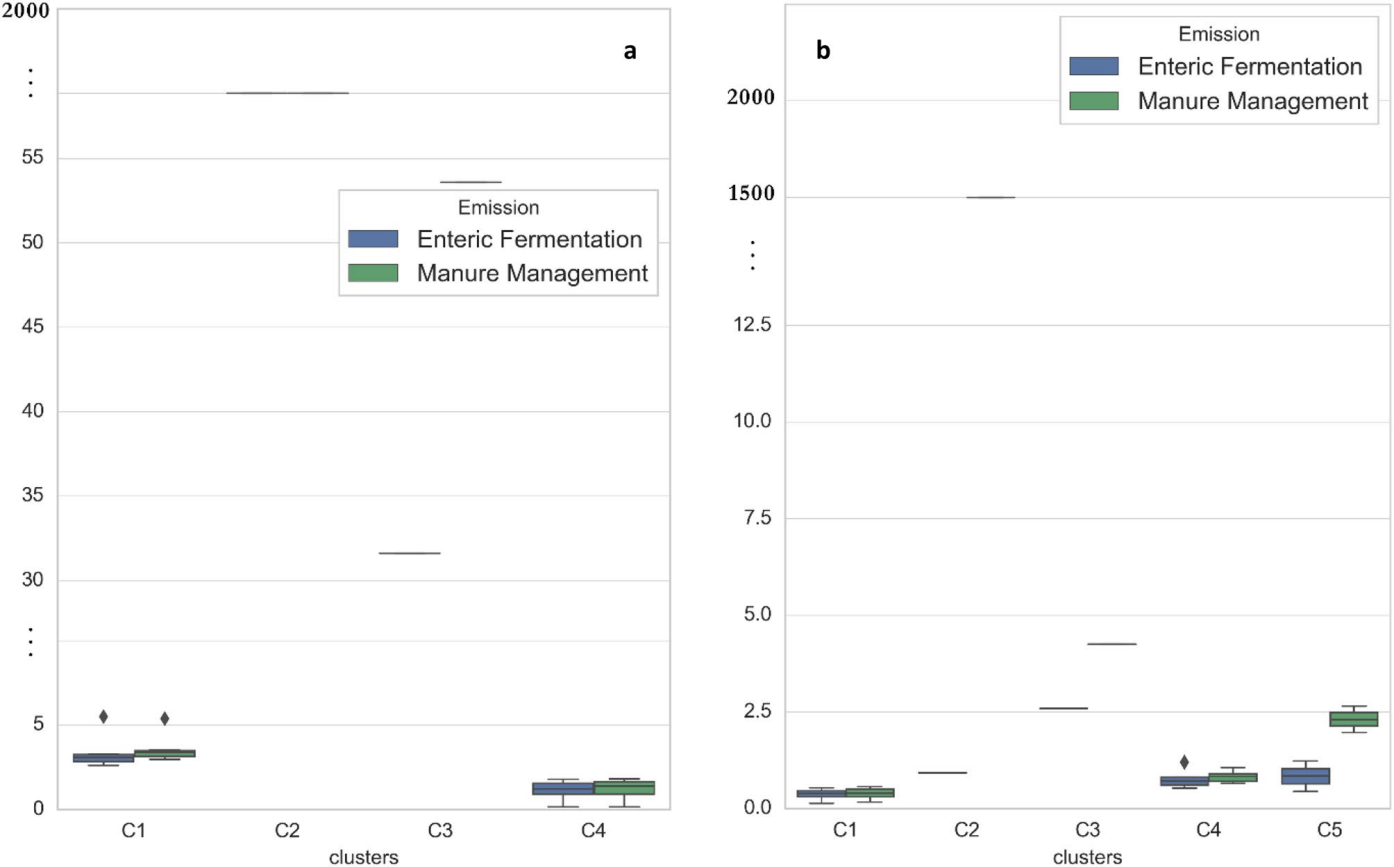
Ratio of CH_4_ emissions change (enteric fermentation and manure management) between the years 1989 to 2005 (**a**) and 2005 to 2021 (**b**). One indicates to no change. The change was calculated by dividing the values of the recent year by the old year (2005/1989 and 2021/2005); C1–C5: clusters of governorates indicated by Fig. 9

In Fig. 10, these clusters are plotted in boxplot graphs for a detailed examination of the pattern of each cluster. The first division (2005/1989) showed a significant change between the emissions of the two years. Cluster C1 displayed a 2.6 to 5.5 times increase in emissions from both enteric fermentation and manure management, while Cluster C3 demonstrated a substantial increase, with enteric fermentation and manure management emissions increasing by factors of 31.6 and 63.6, respectively. Cluster C2 (Luxor) exhibited an extreme increase in both enteric fermentation and manure management emissions, approaching 1500 times. Finally, Cluster C4 showed fluctuation between insignificant decreases and increases in emissions from 1989 to 2005, with changes ranging from 0.15 to 1.8 times for both processes.

In the second division (2021/2005), a mix of increases and decreases in emissions was observed. Cluster C1 showed a decrease in both enteric fermentation and manure management emissions, ranging from 0.13 to 0.66 times. Cluster C2 (Luxor) revealed a significant increase in manure management emissions, close to 1500 times, while enteric fermentation emissions exhibited an insignificant decline, with a factor of 0.93. Cluster C3 showed a reliable significant increase in both enteric fermentation and manure management emissions, with factors of 2.6 and 4.3, respectively. Conversely, Cluster C4 exhibited insignificant decreases to no changes in both enteric fermentation and manure management emissions, with changes ranging from 0.75 to 1.2. Cluster C5 (Matrouh and North Sinai) demonstrated a doubling of manure management emissions, while enteric fermentation emissions increased by 1.2 and decreased by 0.4 times in Matrouh and North Sinai, respectively.

## Discussion

Livestock serves as a significant source of global methane emissions, contributing up to 30% of anthropogenic methane emissions worldwide^45^. In this study, methane emissions were calculated using Tier 1 methodology, primarily relying on factors such as animal category population, emission factors (EF), and animal waste management systems (AWMS). Consequently, higher populations within specific categories correlated with increased methane emissions, and larger animal categories generally exhibited higher EF values.

In recent years, there has been a growing focus on the contribution of the livestock sector, particularly beef and dairy cattle, to GHG emissions. This is primarily due to methane produced by enteric fermentation, the digestive process in ruminant animals, and emissions associated with manure management practices^46–48^. The methane emissions from enteric fermentation in Egypt (Fig. 3a) closely mirrored the livestock population pattern (Fig. 2a). Buffalo, followed by dairy cattle and non-dairy cattle, were the primary contributors to enteric fermentation emissions, driven by growing demands for milk and meat^49,50^. These categories consistently accounted for the highest shares of enteric fermentation emissions, with buffalo and cattle jointly contributing approximately 45% during the study period, except in the last three years when this figure shifted to 33% and 56%, respectively. The buffalo and cattle population presented approximately 20% during the study period, except in the last three years when this figure shifted to 18% and 36%, respectively. The similarity between buffalo and cattle patterns and total enteric fermentation emissions can be attributed to their population similarity and utilized emission factors^39^. The enteric fermentation emissions of buffalo and cattle matched the Egyptian third national community report till 2012^38^. However, while representing 50% of the total population, sheep and goats contributed a smaller share of enteric fermentation emissions due to their lower EF values^45^.

Methane manure management emissions exhibited a similar pattern to methane enteric fermentation emissions, albeit with lower values (Fig. 3b). Buffalo and cattle contributed around 50% and 40% of manure management emissions, respectively. Poultry initially accounted for approximately 1% of methane emissions from manure management in 1989, increasing to 4% by 2005. However, a significant decline in poultry population occurred in 2006 due to avian flu outbreaks, which struck Egypt in 2006, where over 40 million birds had been culled between 2006 and 2017^51^, leading to a corresponding decrease in methane emissions from manure management. Subsequent increases in poultry populations from 2007 onwards correlated with rising methane emissions from manure management, reaching 15% of total emissions by 2021. This increase can be attributed to rising market demand and the significant increase of buffalo and cattle meat prices compared to poultry, despite declines in buffalo and cattle populations in recent years.

The highest densities of livestock population and methane emissions in Egypt are concentrated in specific governorates, particularly those located in the Nile Delta and agriculturally rich regions. Governorates such as Behera, Sharkia, Kafr El-Sheikh, and Monofia in the Nile Delta, along with Menia, Asyut, Suhag, and Quena in other agriculturally productive areas, exhibit significant concentrations of both livestock populations and methane emissions. The distribution of livestock populations, and the methane enteric fermentation and manure management as well, among governorates in Egypt is intricately linked to various geographic factors (Fig. 8). Regions situated inside the Nile Valley typically exhibit higher populations of buffalo and cattle. This can be attributed to several factors. Firstly, the Nile Valley benefits from more abundant water resources compared to other regions, making it conducive to the husbandry of large ruminants such as buffalo and cattle, which have higher water requirements^52,53^. Additionally, the fertile soils and availability of arable land in the Nile Valley are well-suited for agricultural practices, including the rearing of livestock^54^. Conversely, regions outside the Nile Valley, particularly in arid and semi-arid areas, are often dominated by smaller ruminants such as sheep, goats, and poultry. These areas experience harsher climatic conditions, characterized by limited water availability and higher temperatures. As a result, farming practices in these regions tend to favour livestock species that are more resilient to arid environments and have lower water requirements. Sheep, goats, and poultry are better adapted to these conditions, making them more prevalent in regions outside the Nile Valley. Furthermore, farming practices and cultural traditions also play a significant role in shaping the distribution of livestock populations. Nomadic herding practices, common in arid regions, often involve the rearing of sheep, goats, and camels, which can graze over large distances in search of scarce vegetation and water sources. In contrast, agricultural farming methods, predominant in the Nile Valley, are better suited for the intensive management of larger ruminants such as buffalo and cattle. Overall, the distribution of livestock populations among governorates in Egypt is influenced by a combination of factors, including water availability, climatic conditions, and farming practices. Understanding these dynamics is essential for effective livestock management and resource allocation across different regions of the country.

The linear trends in methane emissions across Egyptian governorates generally indicate increasing trends over the study period. However, certain governorates, such as Kafr El-Sheikh and Red Sea, exhibit significant declines in methane emissions. These declines can be attributed to decreases in livestock populations within these regions, which may result from various factors such as changes in agricultural practices, economic conditions, or environmental factors. Conversely, other governorates experience increases in methane emissions driven by population growth and expansion of livestock farming activities. These increases may be influenced by factors such as rising demand for livestock products, agricultural expansion, or changes in land use practices.

Despite variations in methane emissions trends among governorates, cluster analysis did not reveal distinct regional clustering patterns. This suggests that changes in emissions levels were not necessarily dependent on specific geographic or climatic boundaries. This is significant because it challenges the assumption that mitigation strategies can be uniformly applied based solely on regional boundaries. Instead, these fluctuations in methane emissions are likely influenced by various factors. Livestock management practices, including feeding approaches, methods of manure management, and decisions regarding animal breeding, are known to exert significant effects on methane emissions^47,55^. Economic conditions, such as feed costs and the prices of livestock products, can similarly affect these practices^56^. Furthermore, governmental policies concerning agricultural subsidies or environmental regulations may contribute to shaping methane emissions^57^. These variables can exhibit significant variation across various regions within Egypt. Understanding these dynamics is crucial for developing targeted mitigation strategies to reduce methane emissions from the livestock sector while ensuring sustainable livestock production practices across the country. A uniform strategy may not yield optimal results for all regions. For example, regions characterized by intensive dairy production may derive greater benefits from interventions aimed at enhancing manure management practices^58^. Conversely, areas with extensive grazing systems may necessitate strategies focused on optimizing feed quality to effectively address methane emissions^58^. Furthermore, ensuring the sustainability of livestock production practices is paramount^58,59^. Mitigation efforts should not come at the expense of animal welfare or farmer livelihoods. Implementing incentive programs or knowledge-sharing initiatives can encourage farmers to adopt best practices that are both environmentally friendly and economically viable.

### Limitations of the study

Due to the long study period and large geographical area, only Tier 1 methodology was used for calculation, leading to uncertainties in the estimation of greenhouse gas emissions^60^. Factors such as animal size variation, feed quality, and waste management system development further contributed to uncertainty. Additionally, the difficulty in estimating the development of animal waste management systems during the studied period affected methane emissions from manure management.

### Mitigation strategies

After estimating methane emissions from Egypt's livestock sector, suggesting mitigation options is crucial. The nation's Third National Communication outlines formal strategies aimed at enhancing livestock production while adapting to climate change^61^. These strategies include adjusting stocking densities, integrating livestock and crop systems, and employing supplementary feeds. Alternative mitigation options proposed by researchers include technological interventions, nutritional strategies, and pasture management improvements. These additional strategies supplement formal measures and offer a comprehensive approach to addressing methane emissions from the livestock sector^62^. Additionally, animal breeding offers a compelling long-term approach with the potential for sustained reductions^63^. Egypt is actively pursuing strategies to improve animal productivity as a means of mitigating greenhouse gas emissions, particularly methane, from the sector. This focus on productivity enhancements aligns with the concept of "sustainable intensification." Examples of such productivity improvements include the introduction of high-yielding breeds, the implementation of better feeding practices, and advancements in animal healthcare. These efforts hold promise for achieving a more environmentally responsible livestock sector in Egypt.

## Conclusion

In this study, we conducted a comprehensive analysis of livestock methane emissions in Egypt from 1989 to 2021, considering the variations across governorates inside and outside the Nile Valley. Utilizing 2019 refinement of IPCC Guidelines 2006 for National GHG Inventories equations and EFs, we estimated methane emissions from enteric fermentation and manure management, revealing dynamic trends over the study period.

Our findings indicate a fluctuating pattern of methane emissions from livestock, with distinct phases observed from 1989 to 2021. Initially, emissions showed variability from 1989 to 1998, followed by a persistent increase from 1999 to 2018, and a sharp decline between 2019 and 2021. Despite a reduction in livestock census to around half of the population, total methane emissions decreased from 9736.4 Gg CO_2e_ yr^−1^ in 1989 to 7638.2 Gg CO_2e_ yr^−1^ in 2021, with the highest emissions recorded in 2012 at 16,774.8 Gg CO_2e_ yr^−1^. Notably, manure management emissions accounted for about 5 to 6% of the total methane emissions. Livestock methane emissions inside the Nile Valley dominated the overall emissions, starting at around 86% in 1989, peaking at 95% during the period 2000–2010, and stabilizing at 91% in 2021. This trend aligns with the higher population density inside the Valley, driven by better availability of green fodder and favorable conditions for milk production. In contrast, livestock populations outside the Valley, particularly sheep, goats, and poultry, contributed significantly to emissions, reflecting variations in water availability, climatic conditions, and farming practices.

Governorates with the highest livestock populations and methane emissions were concentrated in the Nile Delta, including Behera, Sharkia, Kafr El-Sheikh, and Monofia, as well as Menia, Suhag, and Quena in Upper Egypt. These regions are agriculturally rich, with favorable conditions for livestock rearing and intensive agricultural practices. Understanding the spatial distribution of emissions and the underlying drivers is essential for playing a pivotal role in shaping emissions patterns at the national level and formulating targeted mitigation strategies tailored to specific regions and livestock categories.

Moving forward, efforts to improve data collection and enhance the accuracy of national GHG inventories should be prioritized. By adhering to best practices outlined by international guidelines such as those provided by the IPCC, policymakers can better assess emissions trends and develop effective mitigation strategies. Collaborative initiatives involving stakeholders across the livestock sector are essential for implementing sustainable practices that reduce emissions while ensuring the resilience and viability of livestock production in Egypt. In conclusion, our study underscores the need for continued research and concerted action to address methane emissions from the livestock sector. By leveraging our insights into emissions dynamics and spatial distribution, we can pave the way for a more sustainable and environmentally responsible livestock industry in Egypt.

## Data Availability

The animal population data were collected from the statistical annual books of the Economic Affairs Sector (EAS) and the Central Agency for Public Mobilization and Statistics (CAPMAS) from 1989 to 2021. The used values of EFs were from the 2019 refinement IPCC guidelines 2006. The datasets used and/or analysed during the current study could be available from the corresponding author on reasonable request.
